# α2β1 integrins spatially restrict Cdc42 activity to stabilise adherens junctions

**DOI:** 10.1186/s12915-021-01054-9

**Published:** 2021-06-23

**Authors:** Jake D. Howden, Magdalene Michael, Willow Hight-Warburton, Maddy Parsons

**Affiliations:** Randall Centre for Cell and Molecular Biophysics, King’s College London, New Hunts House, Guys Campus, London, SE1 1UL UK

**Keywords:** Epithelial cells, Integrins, E-cadherin, Beta-catenin, Cytoskeleton, Cell–cell adhesion, Cdc42, RhoGDI, Migration, Proliferation

## Abstract

**Background:**

Keratinocytes form the main protective barrier in the skin to separate the underlying tissue from the external environment. In order to maintain this barrier, keratinocytes form robust junctions between neighbouring cells as well as with the underlying extracellular matrix. Cell–cell adhesions are mediated primarily through cadherin receptors, whereas the integrin family of transmembrane receptors is predominantly associated with assembly of matrix adhesions. Integrins have been shown to also localise to cell–cell adhesions, but their role at these sites remains unclear.

**Results:**

Here we show that α2β1 integrins are enriched at mature keratinocyte cell–cell adhesions, where they play a crucial role in organising cytoskeletal networks to stabilize adherens junctions. Loss of α2β1 integrin has significant functional phenotypes associated with cell–cell adhesion destabilisation, including increased proliferation, reduced migration and impaired barrier function. Mechanistically, we show that α2β1 integrins suppress activity of Src and Shp2 at cell–cell adhesions leading to enhanced Cdc42–GDI interactions and stabilisation of junctions between neighbouring epithelial cells.

**Conclusion:**

Our data reveals a new role for α2β1 integrins in controlling integrity of epithelial cell–cell adhesions.

**Supplementary Information:**

The online version contains supplementary material available at 10.1186/s12915-021-01054-9.

## Background

Epidermal basal keratinocytes are anchored to the extracellular matrix (ECM) via integrin-mediated focal adhesions and to each other via cadherin-based adhesions, as well as tight junctions and desmosomes [1]. Loss of integrin function leads to skin blistering, highlighting the importance of integrins in epidermal homeostasis [2]. Robust cell–cell contacts are required between cells in the basal layer of the epidermis and are essential in maintaining an intact monolayer. However, cell–cell contacts must also be dynamic to enable cell movement, such as during wound healing after epidermal damage [3], proliferation and cell extrusion. Cell proliferation in the epidermis is essential for renewal of the diversified strata [4] and integrin and cadherin-based adhesion signalling play key roles in regulating this process. For example, increased proliferation in skin cancer has been associated with altered integrin expression and deletion of keratinocyte β1 integrins leads to a hyper-proliferative state in the epidermis [2].

α2β1 integrin is constitutively expressed in keratinocytes and is known to bind to collagens and laminins [5, 6]. Upon ligand-binding, α2β1 integrin becomes activated and triggers a signalling cascade that assists in the formation of focal adhesions at the basal membrane of adherent cells [7]. Whilst this role of integrins in binding to ECM proteins is well-established, α2β1 integrin has also been observed at cell–cell junctions and has been proposed to bind to the extracellular domain of E-cadherin in vitro [8–11]. α2β1 has also been observed at junctions between melanoma cells where it co-localises with cadherins [12], and active α2β1 has been shown to contribute to cell–cell adhesion [13]. However, the function of integrins at cell–cell adhesion sites remains unknown.

Recent advances in proteomics analysis have shown that integrin- and cadherin-based adhesions share some signalling and adapter proteins which contribute to the regulation and balance of the two adhesion types in epithelial monolayers [14]. Both adhesion types are also under the control of the Rho family of GTPases that act to co-ordinate F-actin and microtubule cytoskeletons [15, 16]. We hypothesised that α2β1 integrin may positively contribute to adherens junctions and thus act as a dual player in keratinocyte monolayer integrity. Our data demonstrates that α2β1 integrins indeed act to co-ordinate F-actin organisation at keratinocyte cell–cell adhesions through spatio-temporal suppression of Cdc42 activation and this in turn stabilises the E-cadherin:β-catenin complex. Mechanistically, we show that active α2β1 integrins are required for initial recruitment of the phosphatase SHP2 that attenuates activity of Src and RhoGDI leading to sequestration of active Cdc42 from junctions. These findings highlight a new role for α2β1 integrins at cell–cell adhesions that contributes to epithelial integrity.

## Results and discussion

### α2β1 integrins contribute to cell–cell adhesion assembly

**Fig. 1:**
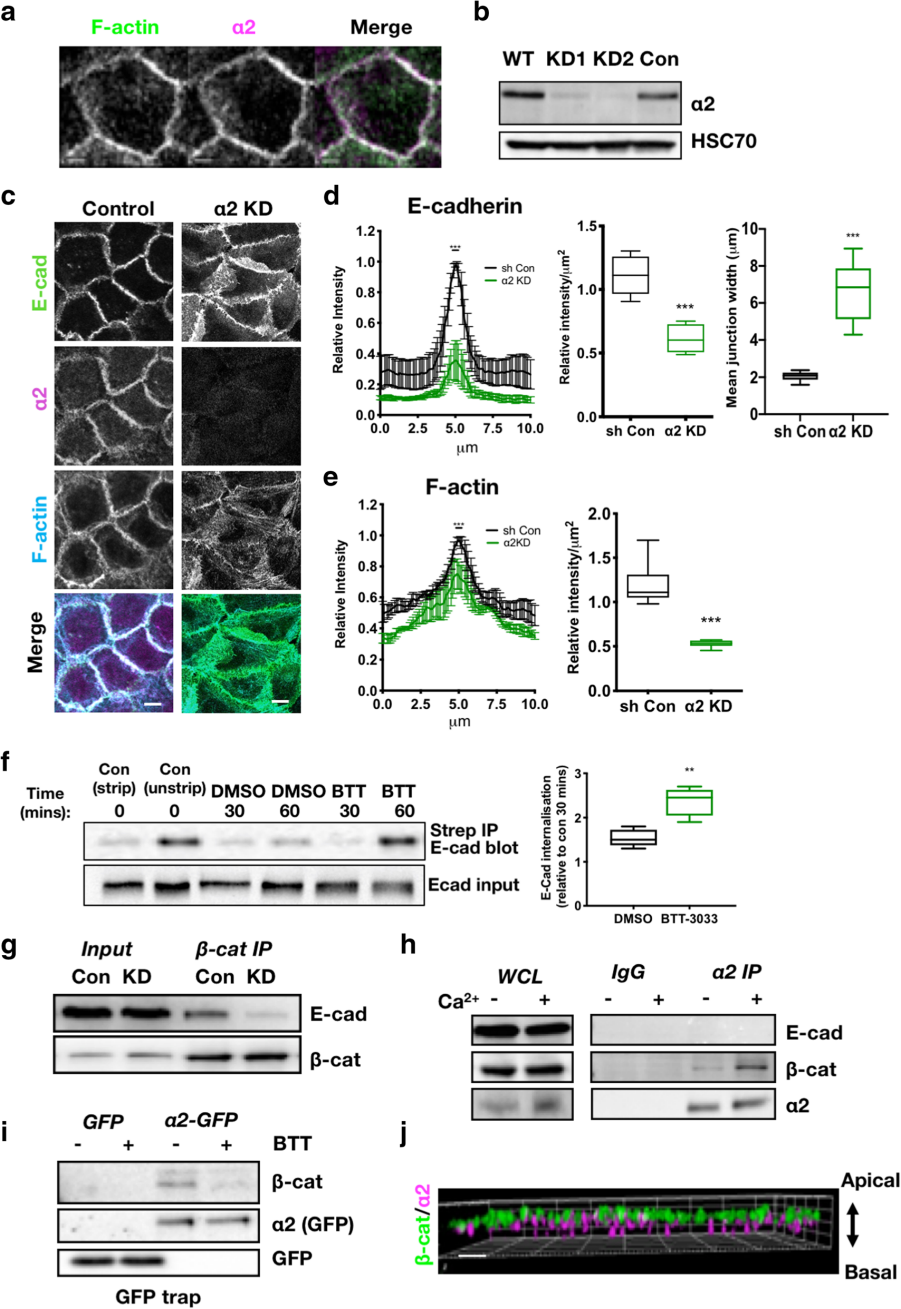
α2β1 integrins are required for stable adherens junctions. **a** Confocal image of normal human keratinocytes (WT) monolayers in 2-mM Ca^2+^ fixed and stained for F-actin and α2 integrin. Scale bar 10μm. **b** Western blot of lysates from WT, control shRNA (Con) and α2 knockdown shRNAs (KD1 and KD2) keratinocytes probed for α2 integrin and HSC70 loading control. **c** Confocal image of control and α2KD keratinocytes stained for E-cadherin (E-cad), F-actin and α2 integrin. Scale bars 10μm. **d** Line graph and quantification of E-cad intensity distribution across junctions, and quantification of peak intensity at junctions from images as in **c**. Data are from at least 30 images per condition over 3 independent experiments. **e** Line graph and quantification of F-actin intensity distribution across junctions from images as in **c**. Data are from at least 30 images per condition over 3 independent experiments. **f** Representative western blot of control and α2KD cells subjected to surface biotinylation and stripping at 30/60 min followed by Streptavidin IP and probing for E-cadherin (top blots). E-cadherin input shown on bottom blot. Graph shows quantification of internalised E-cad from 4 independent experiments (mean+/−SEM). **g** Lysates from control and α2KD cells immunoprecipitated with β-catenin antibodies and complexes probed for β-catenin (β-cat) and E-cadherin. **h** WT cell lysates +/−2mM Ca^2+^ immunoprecipitated with α2 antibodies or control IgG and complexes probed with E-cadherin, β-catenin or α2 integrin antibodies. **i** GFP trap of lysates from α2KD cells expressing GFP or α2-GFP in 2-mM Ca^2+^ treated with DMSO (−) or BTT3033 (BTT; +) and probed for β-catenin and GFP. **j** X,Z reconstructions from structured illumination microscopy (SIM) images of WT junctions in 2-mM Ca^2+^ stained for α2 integrin and β-catenin. Scale bar 1μm. ***p<0.001, **p<0.01

We first confirmed previous reports of α2β1 localisation to cell–cell adhesions in other cell types, by immunostaining normal human keratinocytes. Images demonstrated that α2β1 integrins, but not α3β1 or α5β1integrins, localised to cell–cell adhesions (Fig. 1a; Additional file 1: Fig S1A). We also observed α4β1 integrins at cell–cell adhesions (Additional file 1: Fig S1A). Further analysis of cell monolayers in the presence of high extracellular Ca^2+^ (to induce cadherin-dependent adhesion) demonstrated active β1 integrins were only present at mature cell–cell adhesions and that these integrins were in an active conformation at these sites (Additional file 1: Fig S1B). α2β1 integrins have been suggested to be sensitive to the nature of divalent cations present in the extracellular environment, and particularly that higher levels of Ca^2+^ compared to Mg^2+^ can inhibit α2β1 function in migrating keratinocytes, and vice versa [17]. To determine whether the balance of these two cations altered active β1 integrin levels at lateral adhesions, we treated keratinocyte monolayers with either Ca^2+^ or Mg^2+^ or both cations and quantified active β1 integrins at cell–cell adhesion sites. Our data demonstrated that Mg^2+^ alone was not sufficient to induce lateral active β1 integrins and that adding both Ca^2+^ and Mg^2+^ did not enhance active integrins above that seen with Ca^2+^ alone (Additional file 1: Fig S1C) suggesting Mg^2+^ does not contribute to integrin activity at these sites.

To determine the function of α2β1 integrins at later adhesion sites, we used two different shRNA sequences to stably deplete α2β1 integrins from keratinocytes (Fig. 1b, α2KD) and demonstrated this had no effect on total expression levels of the other integrin subunits or cell adhesion molecules (Additional file 1: Fig S1D). Depletion of α2 integrins resulted in loss of active β1 integrins at cell–cell adhesion sites (Additional file 1: Fig S1E), further suggesting α2β1 constitute a significant proportion of this active β1 pool. To explore whether loss of α2 altered organisation of adhesion components, confocal images of monolayers of control and α2KD cells were stained for F-actin and E-cadherin. Images demonstrated that defined cortical E-cadherin and F-actin at cell–cell adhesion sites in control cells were more disorganised in α2KD cells and junctions were slipped beneath membranes of neighbouring cells (Fig. 1c). Quantification of images revealed a significant reduction in E-cadherin and F-actin intensity at cell–cell adhesions (Fig. 1d, e) and a significant increase in junction width (Fig. 1d) between α2β1 depleted cells, compared to controls.

To determine whether this phenotype was dependent upon active α2β1 integrins, experiments were repeated in cells treated with BTT-3033 (BTT), a small molecule inhibitor to α2β1 that blocks both active and inactive forms of this integrin [18]. Images and analysis revealed that BTT treatment phenocopied the loss of 12G10 staining and junctional integrity seen in α2KD cells (Additional file 1: Fig S1E, F) indicating active and/or ligand-bound integrin is required to stabilise cell–cell contacts. Despite α2β1 integrins being focal adhesion proteins, α2KD or BTT-treated cells showed a significant increase in vinculin-positive cell–matrix adhesions at the basal surface (Additional file 1: Fig S1G) suggesting α2β1 integrins are not required for focal adhesion assembly in keratinocytes. It is notable that we could not detect the intracellular adaptor and integrin activating protein talin at cell–cell adhesions under any conditions analysed (not shown), which may suggest alternative intracellular binding partners are required for integrin activity at junctions, potentially including kindlins which are known to localise to these sites [19]. However, as a previous study has demonstrated a specific 70kDa cleaved form of the C-terminus of talin can be recruited to cell–cell adhesions, it remains possible that this may contribute to integrin activity at these sites [20]. Consistent with our observations of disrupted cell–cell adhesion, further characterisation revealed that α2KD cells exhibited increased monolayer permeability (Additional file 1: Fig S1H), increased proliferation rates (Additional file 1: Fig S1I) and reduced collective migration (Additional file 1: Fig S1J), which were rescued by re-expression of α2-GFP. Our data showing increased proliferation and a defect in wound healing in α2KD cells is in agreement with studies showing α2β1 integrin is a proliferation suppressor and its loss is implicated in cancer metastasis [21]. α2β1 integrin was considered an essential collagen receptor, and it was presumed that its loss would lead to significant developmental defects. However, α2 integrin knockout mice show no obvious phenotypes under homeostatic conditions and no change in re-epithelialisation of skin wounds, but do show reduced angiogenesis in wounds and reduced keratinocyte migration in vitro [22–24]. Conversely, keratinocyte migration and wound closure are dependent on the α2β1 integrin human cells and skin explants [25, 26] suggesting potential discrepancies between human and mouse models.

Adherens junction stability is in part controlled by the rate of E-cadherin internalisation, under the control of p120catenin [27]. To determine whether the observed disruption of E-cadherin at junctions in α2β1-depleted cells correlate with enhanced E-cadherin internalisation, cells were subjected to surface biotinylation in the presence or absence of BTT followed by E-cadherin immunoprecipitation (IP). Data revealed a significant increase in E-cadherin internalisation rates in cells treated with BTT compared to DMSO controls (Fig. 1f). This enhanced internalisation also correlated with reduced E-cadherin:β-catenin complex formation in α2β1-depleted cells (Fig. 1g). To determine whether α2 integrins may exert this effect through formation of a complex with adherens junction proteins, IPs of α2 from cells in low or high Ca^2+^ were probed for E-cadherin and β-catenin. Data demonstrated that β-catenin, but not E-cadherin was in complex with endogenous α2 integrins and with α2-GFP re-expressed in α2KD cells; this was more pronounced in both cases when cells formed mature cell–cell adhesions (Fig. 1h, i). It is also noteworthy that α2 integrins were identified in a recent proteomic analysis of the cadherin adhesome [28], further supporting our observations. Moreover, pre-incubation of WT cells with BTT resulted in a reduction in levels of β-catenin associated with the integrin (Fig. 1i) indicating that ligand-bound α2β1 promotes stabilisation of this complex. Analysis of fixed cells using high resolution structured illumination microscopy (SIM) further demonstrated that α2 integrins were positioned basal to adherens junctions but that a proportion overlapped with β-catenin in the Z-plane (Fig. 1j). This suggests that integrins are positioned basally to adherens junctions but elevated from the basal surface as very little α2 was detected in focal adhesions. Combined, these experiments show that α2 integrins can associate with β-catenin at cell–cell adhesion sites and loss of active α2 leads to dissociation of E-cadherin from the plasma membrane, resulting in de-stabilised adherens junctions. One previous study has reported α3 integrins forming a complex with E-cadherin and β-catenin in kidney epithelial cells [29]. In this case, the complex was proposed to be indirectly controlled through integrin-CD151 tetraspanin association at adherens junctions. α2 integrins have not been reported to form complexes with tetraspanins so a similar mode of association is unlikely for α2, but further experiments would be required to rule this possibility out.

### α2β1 co-localises with laminin at cell–cell adhesions

α2 has previously been suggested to bind E-cadherin in vitro [9]. Our data did not demonstrate a complex between these receptors in cis, but to determine whether loss of α2 impaired E-cadherin-dependent adhesion, we plated control, α2KD or BTT-treated cells on collagen I or Laminin-322 (ligands for α2) or immobilised Fc-E-cadherin ectodomain. A significant reduction in cell adhesion to both ECM proteins was seen in α2KD or inhibited cells, whereas no impairment of binding to fc-ECad was observed (Additional file 2: Fig S2A). Whilst not definitive proof, this data strongly suggests that α2 does not modulate E-cadherin homo-dimerisation and that α2 is highly unlikely to be a receptor for E-cadherin in trans. Notably however, α2 integrins were partially co-located with E-cadherin in cells plated on fc-ECad (Additional file 2: Fig S2B), in agreement with images in intact cell monolayers and suggesting E-cadherin engagement is sufficient to recruit α2 to these sites of homo-dimerisation, possibly through association with β-catenin.

**Fig. 2:**
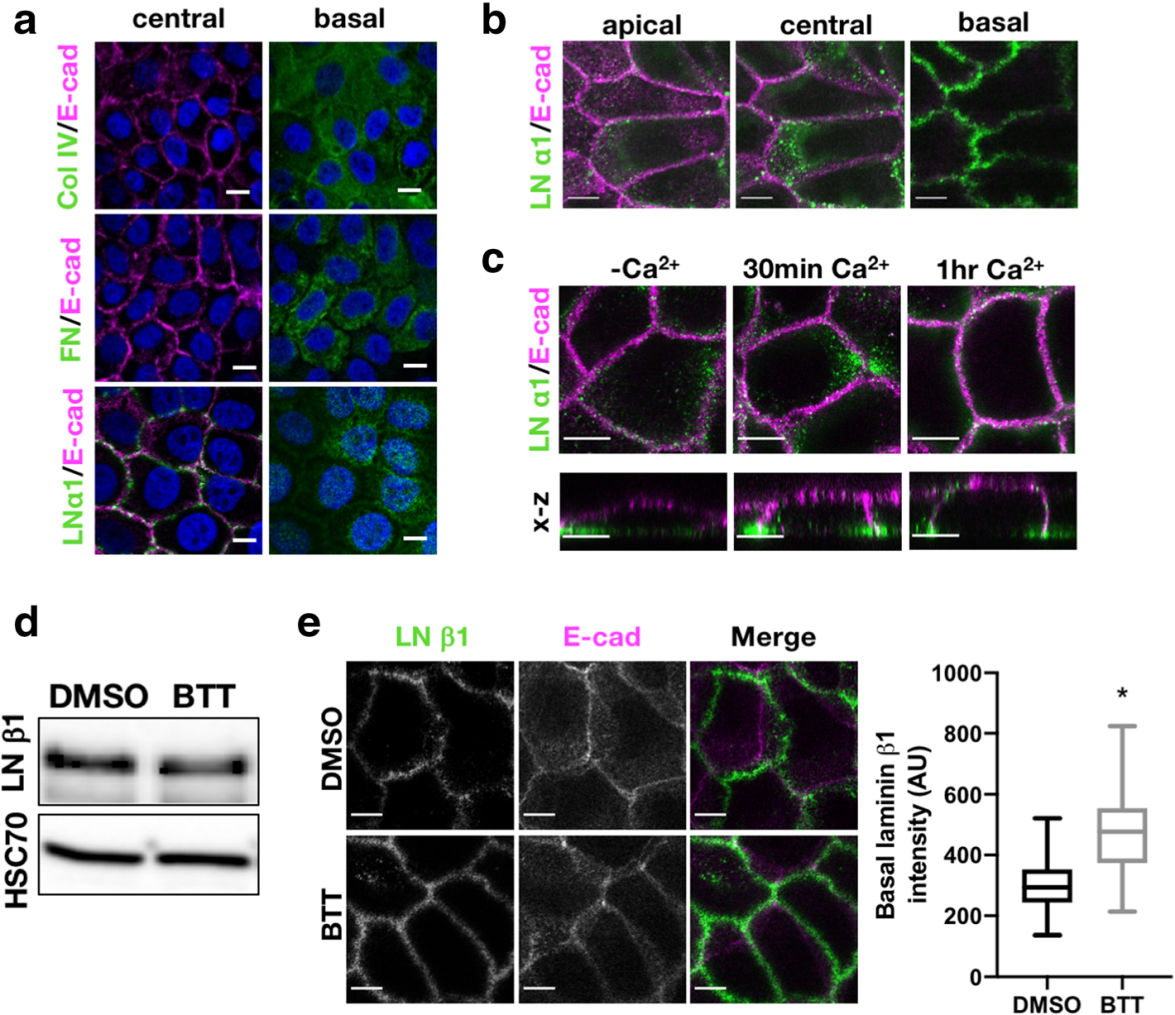
Laminin localises to cell–cell adhesions. **a** Confocal images of central and basal planes of WT monolayers in 2-mM Ca^2+^ fixed and stained for laminin α1, collagen IV or fibronectin. **b** Confocal slices of apical, central and basal planes of WT monolayers in 2mM Ca^2+^ stained for laminin α1 and F-actin or E-cadherin. **c** Images of the apical plane and x-z images of DMSO- and BTT (20 μm, 1h)-treated monolayers in 2-mM Ca^2+^ fixed and stained for laminin α1 and E-cadherin. **d** Western blot of laminin β1 in DMSO- and BTT-treated keratinocytes. **e** Basal laminin β1 intensities and quantification. Data are means −/+ SEM from at least 30 images per condition over 3 independent experiments; *p<0.05. Scale bars, 10μm throughout

In order to determine whether ECM proteins were present at cell–cell adhesion sites and may act as α2 ligands in this context, WT cell monolayers were fixed and stained for collagen IV, fibronectin or Laminin β1 or α3 chains. Confocal Z-stack analysis revealed an accumulation of laminin at cell–cell adhesions, whereas both fibronectin and collagen IV were located at the basal cell surface (Fig. 2a, b). Laminin accumulation at junctions appeared rapidly after induction of adherens junction formation, indicating a potential active secretion or positioning of this ECM protein to these sites (Fig. 2c). Furthermore, laminin localisation was reduced at cell–cell adhesion sites and increased at the basal surface of cells treated with BTT, without any change in total laminin levels, indicating that active α2, or stable adherens junctions, are required for lateral laminin positioning (Fig. 2d, e; Additional file 2: Fig S2C). This data demonstrates that active α2 integrins co-locate with laminins at cell–cell adhesions and suggest laminin may act as a ligand for this integrin at these sites. Recent studies have also identified ECM components at junctional adhesions in vivo including ligands for α2β1 and α4β1 [30–32]. To determine whether may also be the case in skin, we stained human skin sections for α2, Laminin or Collagen IV. α2 was located to lateral adhesion sites between basal keratinocytes (Additional file 2: Fig S2D) in agreement with our in vitro data and as others have previously shown [8, 26]. Laminin staining was also predominantly basal but was more convoluted in appearance with some inter-digitation with the epidermis as compared to collagen IV (Additional file 2: Fig S2D). We also note with interest that such inter-digitation has been observed in other human skin sections [33] and cornea [34]. It is also notable that other ECM ligands may play a role in this context, including collagen XXIII that has been shown to be a ligand for α2β1 and localises to cell–cell adhesions in the human skin [35]. Whilst this possible recruitment of ECM ligands between cells remains poorly understood, this is suggestive of conserved positioning of integrin ligands within different epithelia that may provide additional strength to these tissues.

### α2β1 integrins temporally co-ordinate Cdc42 activity at cell–cell adhesions

**Fig. 3:**
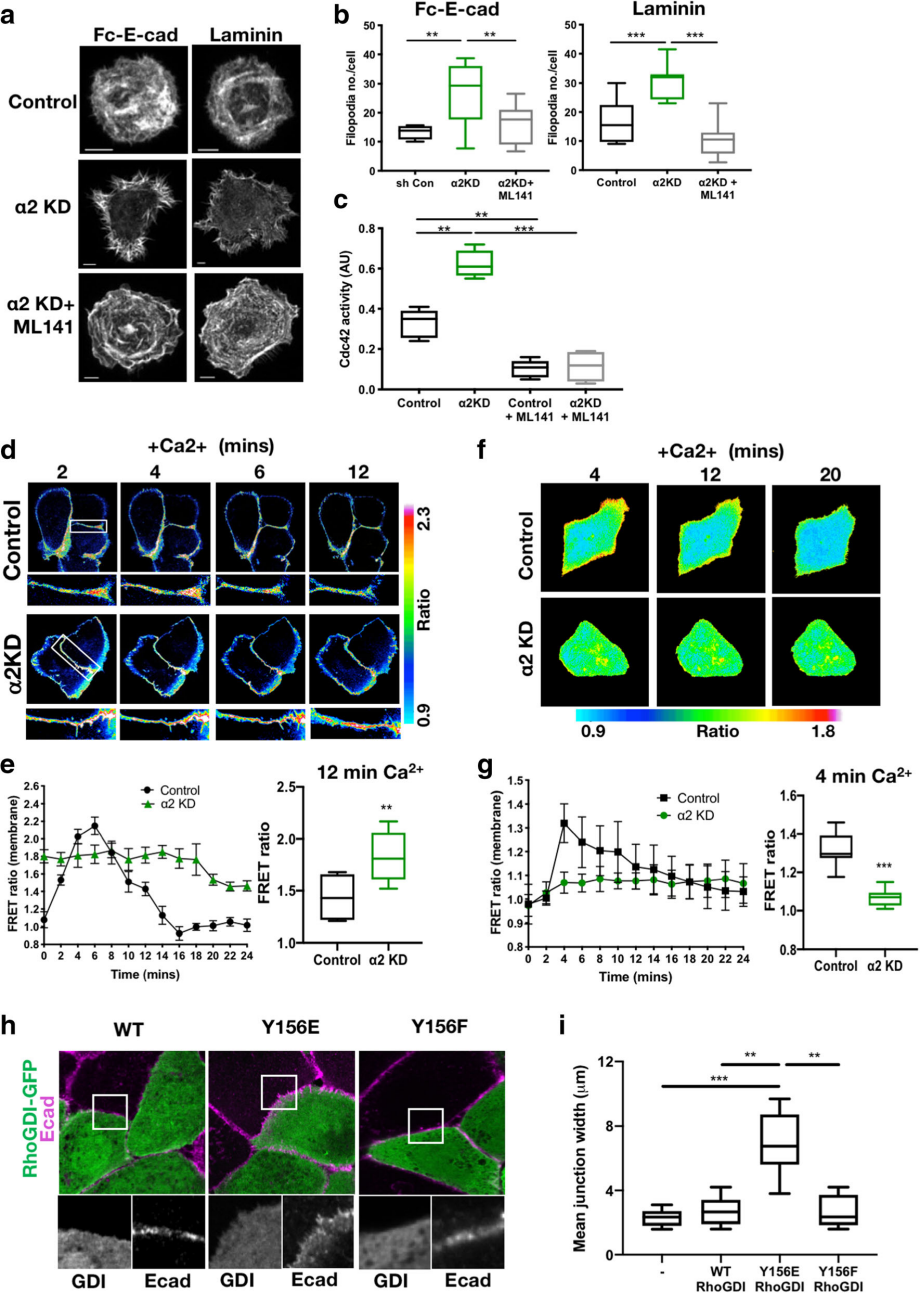
α2 integrins suppress Cdc42 activity upon cell–cell adhesion formation. **a** Representative images of control, α2 knockdown (KD) and α2KD cells pre-treated with ML141 (10 μM, 4h) plated onto laminin or Fc-E-cadherin-coated coverslips for 30 min and fixed and stained for F-actin. **b** Quantification of the number of filopodia per cell for cells adhered to laminin or Fc-E-cadherin. Data are from at least 30 cells per condition; representative of 3 independent experiments. **c** Analysis of Cdc42 activity in cell lysates from control and α2KD monolayers in 2-mM Ca^2+^ treated with either DMSO or ML141 (10 μM, 4h) by G-LISA. N=3 wells per condition; representative of 3 independent experiments. **d** FRET/donor ratiometric images of control and α2KD cells expressing the Cdc42 FRET biosensor taken from movies following a Ca^2+^ addition time course. White boxes highlight cell–cell junctions. **e** Quantification of the relative changes in Cdc42 FRET/donor ratios at junctions over time. Data are means −/+ SEM from 18 cells pooled from 3 independent experiments. **f** FRET/donor ratiometric images of control and α2KD cells expressing the Cdc42 GDI FRET biosensor taken from movies following a Ca^2+^ addition time course. **g** Quantification of the relative changes in GDI FRET/donor ratios within cells over time. **h** Images of RhoGDI-GFP and Y156 phosphomutants (Y>E and Y>F) expressed in WT cells, fixed and stained for E-cadherin. **i** Quantification of mean E-cadherin junction width from cells as in **h**. Scale bars, 10μm throughout. P values = ***p<0.001, **p<0.01

Co-ordination of F-actin organisation is required for assembly and maintenance of cell–cell adhesions, and this requires precise regulation of members of the family of Rho GTPases. Previous studies have shown that Rac1 and Cdc42 both promote protrusions at cell–cell contacts to promote E-cadherin-based adhesion [36]. As junctions mature, F-actin becomes more linear and exerts tension via the actomyosin machinery which helps to maintain linear F-actin bundles parallel to cell–cell contacts [37]. RhoA activity is downregulated on initial junction formation and then increases once robust cell–cell contacts develop to support cortical F-actin [38, 39]. Cdc42 promotes filopodia assembly and is activated on initial cadherin contact and junction formation and is downregulated with maturation [40]. This downregulation is necessary as Cdc42 also promotes internalisation of E-cadherin and disrupts junction formation [41]. To understand whether α2β1integrins may be co-ordinating cytoskeletal arrangements to promote adhesion maturation, we analysed F-actin organisation in control and α2KD cells plated on fc-ECad or laminin. We observed a striking increase in assembly of filopodia in α2β1-depleted cells plated on both ligands (Fig. 3a) that was confirmed by formal quantification of filopodia number (Fig. 3b). As filopodia are associated with Cdc42 activity, we analysed global activation of this GTPase in control and α2KD cells. Data revealed a significant increase in Cdc42 activity in α2KD cells, which was reversed by treatment of cells with the Cdc42 inhibitor ML141 (Fig. 3c). Similar findings were observed in cells treated with BTT (not shown). ML141 significantly reduced filopodia formation in cells plated on both ligands (Fig. 3b) and also partially restored the appearance of E-cadherin adhesions in α2β1-depleted cells to those seen in control cells (Additional file 3: Fig S3A, B). These data demonstrate that enhanced active Cdc42 in α2β1-depleted cells contributes to enhanced filopodia assembly and subsequent defects in adherens junction formation upon initial cell–cell contact.

To further define the α2β1-dependent effect of spatial Cdc42 activity, we imaged control or α2KD cells expressing a Cdc42 FRET biosensor before and after addition of Ca^2+^ to initiate cadherin dependent adhesion. Resulting movies and analysis demonstrated Cdc42 activity increased in control cells 2–8 min following Ca^2+^ addition, and this then reduced over the following 20-min imaging period, consistent with previous studies [40]. However, Cdc42 activity was maintained at higher levels in α2KD cells throughout the imaging period (Fig. 3d, e), confirming that α2 integrins suppress Cdc42 at cell–cell adhesions. Previous studies have shown that integrins at focal adhesions in migrating cells can indirectly activate Cdc42 to promote migration [42, 43]. As our data suggest that α2 at cell–cell adhesions has the opposite effect on Cdc42 activity, this raised the possibility that integrins within this lateral environment may support distinct signalling pathways to control local GTPase activity.

### α2β1 integrins promote RhoGDI activity

To explore how α2 integrins modulate Cdc42 activity, we first analysed whether they may be part of the same complex. IP of α2-GFP from α2KD cells re-expressing this construct demonstrated no detectable Cdc42 in complex with this integrin (Additional file 3: Fig S3C). Activity of GTPases are spatially and temporally controlled by guanine nucleotide exchange factors (GEFs), activating proteins (GAPs) and dissociation inhibitors (GDIs) [44]. Of these, IQGAP1, RhoGDI, RacGAP1 and Tuba have been proposed to localise to cell–cell adhesions and control Cdc42 activity [45–47] and RhoGDI has been shown to bind to integrins [48]. We were unable to detect a complex between α2 integrins and any of these regulatory molecules, and IQGAP, Tuba and RacGAP1 showed no clear change in localisation in α2KD cells (Additional file 3: Fig S3D and not shown). However, analysis revealed a significant increase in endogenous levels of Y156 phosphorylated RhoGDI (pY156) at cell–cell adhesions in BTT treated and α2KD cells (Additional file 3: Fig S3E). Previous studies have shown phosphorylation of Y156 by the non-receptor tyrosine kinase Src results in enhanced GDI-membrane recruitment and reduced suppression of GTPase activity [49], and we confirmed this Src-dependent phosphorylation of RhoGDI also occurred in keratinocytes (Additional file 3: Fig S3F). To determine whether the enhanced RhoGDI Y156 phosphorylation in α2KD cells resulted in attenuation of GDI activity, we performed live imaging of cells expressing a FRET antenna probe that reports on dynamics of GDI–Cdc42 interactions [50]. Data revealed a significant increase in FRET in control cells after Ca^2+^ addition, which rapidly declined back to baseline over the imaging period (Fig. 3f, g) and this correlated with an increase in Tyr-phosphorylated RhoGDI (Additional file 3: Fig S3G). Conversely, α2KD cells showed a constant and consistently lower level of GDI-Cdc42 FRET (Fig. 3f, g) demonstrating that the induction of GDI–Cdc42 binding upon formation of cadherin-based adhesions requires active α2 integrins.

To further determine whether phosphorylation of RhoGDI played a role in junctional stability, we overexpressed either WT or Y156E (phospho-mimic) or Y156F (phospho-dead) form of GDI-GFP in monolayers of cells and analysed E-cadherin organisation. Quantification of images revealed that overexpression of Y156E RhoGDI was sufficient to disrupt cell–cell adhesions, resulting in a significant increase in junction width (Fig. 3h, i) similar to that seen in α2KD cells. Collectively, these findings demonstrate that α2 integrins are required to increase RhoGDI activity at cell–cell adhesions and suppress Cdc42 activity to enable junction maturation. RhoGDI also binds to Rac and RhoA GTPases which are also involved in maintenance of adherens junctions [51]. We analysed RhoA and Rac activity in live cells and saw no differences between control and α2KD cells over the initial period of junction assembly (not shown). This would suggest that RhoGDI preferentially binds to Cdc42 to sequester this GTPase from the membrane following initial contact formation. This may be due to higher concentrations of membrane-associated Cdc42 at this time, or distinct spatial segregation of GTPase species that permits GDI accessibility. Similarly, distinct spatio-temporal control of RhoA, Rac and Cdc42 has been shown to occur at the leading edge of single migrating cells [50, 52], raising the possibility that such co-ordination also exists under the control of both integrins and cadherins at cell–cell adhesions.

### α2 integrins control junctional Src and SHP2 activity

**Fig. 4:**
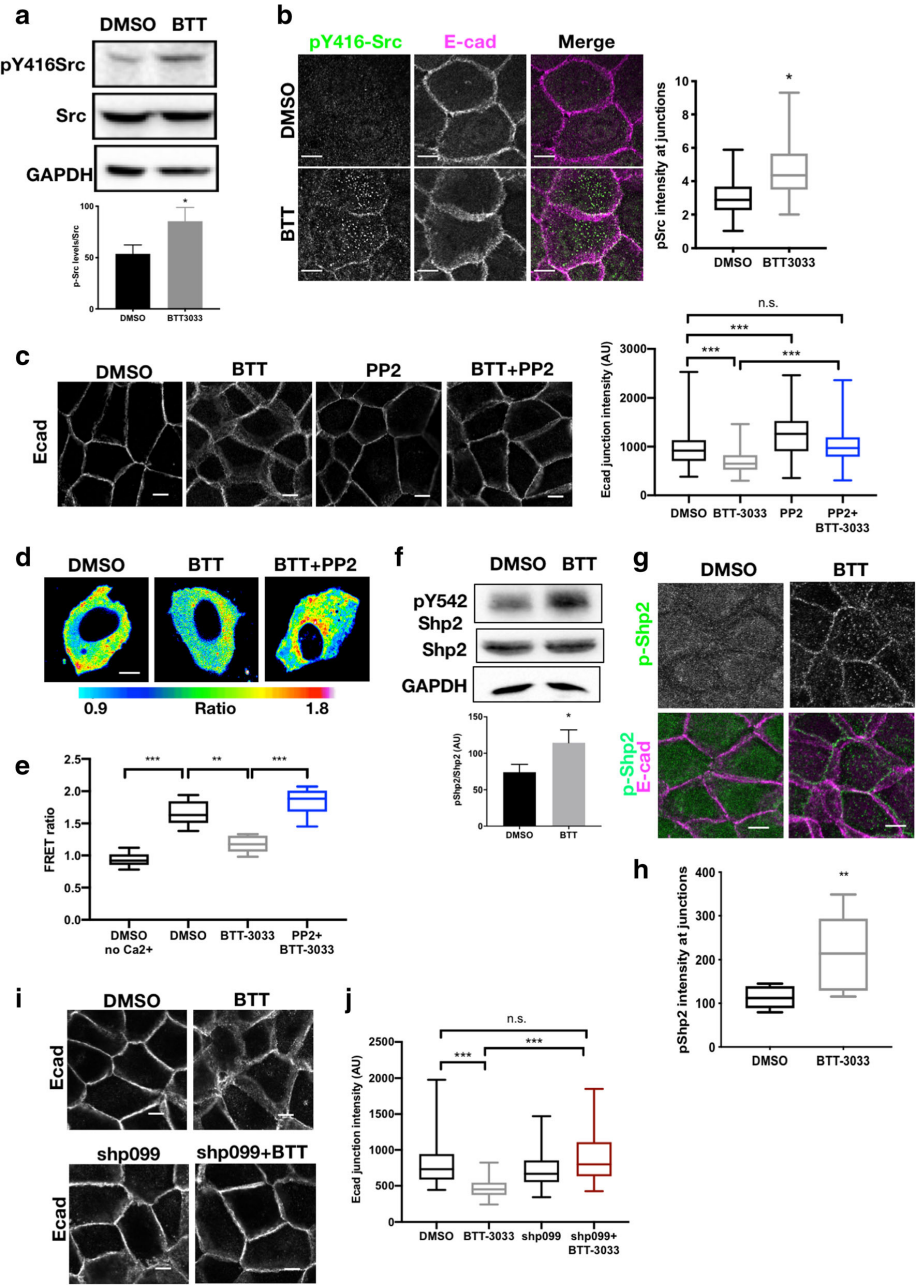
α2 integrins suppress Src and Shp2 activity to activate RhoGDI at junctions. **a** Western blot of WT monolayers in Ca^2+^ treated with either DMSO or BTT (20 μm, 1h) probed for pY416-Src and total Src and quantification from 4 independent experiments. **b** Images of WT monolayers in Ca^2+^, treated with either DMSO or BTT (20 μm, 1h), fixed and stained for p-Src and E-cadherin and quantification of junctional p-Src. Data are from at least 30 images per condition and over 3 independent experiments. **c** Images of WT monolayers in Ca^2+^ , treated with either DMSO or BTT (20 μm, 1h) and/or PP2 (1 μm, 1h), fixed and stained for E-cadherin and quantification of junctional E-cadherin. Data are from at least 30 images per condition and over 3 independent experiments. **d** FRET/donor ratiometric images of live control and α2KD cells expressing the Cdc42 GDI FRET biosensor with or without BTT (20 μm, 1h) or PP2 (1 μm, 1h) treatment. **e** Quantification of FRET ratio levels from cells as in **d**. Data are from at least 18 images per condition and over 3 independent experiments. **F** Western blot of WT monolayers in Ca^2+^ treated with either DMSO or BTT (20 μm, 1h) probed for pY542-Shp2 and total Shp2 and quantification from 4 independent experiments. **g** Images of WT monolayers in Ca^2+^, treated with either DMSO or BTT (20 μm, 1h), fixed and stained for pY542-Shp2 and E-cadherin. **h** Quantification of junctional pY542-Shp2 as in **g**. Data are from at least 35 images per condition and over 3 independent experiments. **i** Images of WT monolayers in Ca^2+^ , treated with either DMSO or BTT (20 μm, 1h) and/or shp099 (1 μm, 1h), fixed and stained for E-cadherin. **j** Quantification of junctional E-cadherin as in **i**. Data are from at least 30 images per condition and over 3 independent experiments. Scale bars 10μm throughout.; n.s. not significant, ***p<0.001, **p<0.01, *p<0.05

As RhoGDI phosphorylation played a critical role in cell–cell adhesion maturation, we explored whether α2 integrins may be regulating this through local control of kinases or phosphatases at cell adhesion sites. We firstly analysed activity of Src, the kinase for RhoGDI Y156. Analysis of active Src (Y416) levels both in whole cell lysates and by confocal imaging revealed a significant increase in active Src at cell–cell adhesions, but not cell–ECM adhesions in both α2 inhibited and depleted cells (Fig. 4a, b and Additional file 4: S4A). Moreover, suppression of Src activity by treating cells with PP2 resulted in a rescue of E-cadherin organisation in α2 inhibited and KD cells (Fig. 4c and Additional file 4: S4B) indicating a key role for α2-dependent suppression of active Src in maintaining junction integrity. Inhibition of Src also significantly increased Cdc42–GDI FRET in α2-inhibited cells (Fig. 4d, e) further supporting the notion that α2 integrins suppress GDI activity through suppression of Src-dependent GDI phosphorylation. Src activity is known to be enhanced through integrin-ECM engagement at focal adhesions in a FAK-dependent manner, leading to control of GTPase activity [53]. Conversely, our data demonstrates that α2 integrins at cell–cell adhesions suppress Src activity, indicating an alternative mechanism exists at cell–cell contacts, potentially through phosphatase-dependent control of Src activation. It is also notable that active Src promotes E-cadherin endocytosis and disruption of epithelial integrity [54, 55] which may provide a further explanation for the α2-dependent phenotypes we document here. A key regulatory phosphatase for Src is Shp2, which dephosphorylates Src Y527 leading to enhanced Src activity [56] and has been shown to bind to integrins [57]. Analysis of localisation of Shp2 revealed significantly higher active (pY542) Shp2 levels at cell–cell adhesions and in lysates of α2 integrin inhibited cells (Fig. 4f–h). Moreover, blocking Shp2 activity resulted in rescue of E-cadherin organisation at cell–cell adhesions in α2-inhibited cells (Fig. 4i, j) and similar to that seen in cells treated with Src inhibitor (Fig. 4c). Taken together, this data demonstrates that α2 integrins suppress activity of Src and Shp2 at cell–cell adhesions leading to enhanced Cdc42–GDI interactions and stabilisation of junctions between neighbouring epithelial cells.

## Conclusions

In conclusion, our study provides previously unreported evidence that active α2β1 is present at epithelial cell–cell adhesions and co-ordinates activity of Cdc42 to enable stabilisation of E-cadherin complexes in trans. This appears to be the dominant role for α2β1 in keratinocytes, as focal adhesion assembly was not reduced upon α2 depletion or inhibition. Although our work reveals a new functional role for α2β1 integrins at these sites, the observed localisation of integrins to cell–cell adhesions in many previous studies suggests our findings hold relevance to other epithelial cell types. Future studies aimed at dissecting the integrin-associated adhesome components at cell–cell adhesions in other models, and cell types will provide means to determine conservation of this role for integrins and how this is balanced between cell–cell and cell–ECM adhesions.

## Methods

### Antibodies and reagents

Anti-α2-integrin (sc-74466), anti-β-Catenin (sc-59737) and anti-RhoGDI (sc-373724) antibodies were from Santa Cruz Biotechnology. Anti-β1-integrin (AB1952), anti-active-β1-integrin (12G10), anti-α3-integrin (MAB2290), anti-p-Src (Y418; 07-909), anti-Src (GD11), anti-GAPDH (MAB374) and anti-GFP (MAB1083) antibodies were from Merck. Anti-p-Shp2 (Y542; 3751) and Shp2 (3752) antibodies were from cell signalling. Anti-E-cadherin (ab1416) and anti-laminin-β1 (ab44941) antibodies were from Abcam. Anti-vinculin (hVIN-1), anti-phosphotyrosine (4G10) and anti-HSC-70 (N69) antibodies were from Sigma-Aldrich. Anti-α4 integrin (MAB1354) was from R&D Systems. Anti-α5-integrin (eBioSAM-1) antibody was from eBioscience. Anti-p-RhoGDI (Y156; OAA100735) antibody was from Aviva Systems Biology. Anti-laminin-α3 (MAB21441) was from Novus Biologicals. Anti-Cdc42 (ACD03) antibody was from cytoskeleton. Anti-mouse horseradish peroxidase (HRP) and anti-rabbit HRP were from Dako. Anti-mouse Alexa 488 and Alexa 568, anti-rabbit Alexa 488 and Alexa 568, Sulfo-NHS-LC-biotin and phalloidin Alexa 647 were all obtained from Thermofisher. BTT-3033, PP2 and ML141 were from Tocris/Biotechne (Bristol, UK); shp099 was from Millipore. α2-integrin and control shRNA vectors were from Sigma-Aldrich. Calyculin A and protease inhibitor cocktail 1 were obtained from Calbiochem. Puromycin was obtained from GE Healthcare.

### Plasmids

Human α2-integrin was amplified using PCR primers (5′ATAGATCTATGGGGCCAGAACGGACAGG-3′ (forward) and 5′-GTCTCGAAGGTGGCGATGGATCCCG-3′ (reverse)) and cloned into a lentiviral pLNTsffv-GFP backbone (a gift from Dr. James Monypenny, King’s College London), between XhoI and MluI sites. Cdc42 Rho biosensor was a gift from M. Matsuda (Osaka University, Japan [58];), and RhoGDI biosensor was a gift from K. Hahn (University of North Carolina, USA [50];). Recombinant Fc-ECad expression plasmid was a gift from J. Nelson (Stanford University, USA). GDI-GFP was a kind gift from N. Saito (Kobe University, Japan [59];); Y156 mutations were introduced into this plasmid using site-directed mutagenesis (QuickChange II, NEB).

### Cell culture

Immortalised Normal Human Keratinocytes were grown in high glucose Dulbecco’s modified Eagle’s medium (DMEM, Sigma-Aldrich) in a 3:1 ratio with Ham’s F12 (Sigma-Aldrich) supplemented with 10% foetal calf serum (FCS, Sera Laboratories International) and RM+ supplement (40 μg/ml Hydrocortisone (Sigma-Aldrich), 500 μg/ml insulin (Sigma-Aldrich), 1 μg/ml EGF (PeproTech), 0.84 μg/ml cholera toxin (Sigma-Aldrich), 500 μg/ml transferrin (Sigma-Aldrich), 1.3 μg/ml lyothyronine (Sigma-Aldrich) and cultured at 37°C in 5% CO_2_. Cells expressing shRNA to target α2-integrin were maintained in DMEM as above puromycin (0.7 μg/ml). HEK293 packaging cells were used to generate lentiviral particles for viral transduction as previously described [60]. Transfections were carried out using Attractene in accordance with the given protocol (Qiagen).

### Immunostaining and microscopy

Keratinocytes with or without 2mM CaCl_2_ (denoted as Ca^2+^) or MgCl_2_(denoted as Mg^2+^) as indicated were fixed after no treatment or treated with DMSO, BTT3033 (20μM), ML141 (10μM). The cells were fixed with 4% paraformaldehyde (PFA) for 10 min and permeabilized with 0.1% Triton X-100. Cells were incubated with primary antibodies for 2 h and with appropriate secondary antibodies conjugated to Alexa Fluor 568 or 488 (1:1000) and phalloidin conjugated to Alexa Fluor 647 (1:500), including Hoechst, for 1 h. Cells were mounted on slides using FluorSave (ICN). Normal human skin sections were processed for staining as previously described [61]. Confocal microscopy was performed using a Nikon A1R inverted confocal laser scanning microscope with a 60x oil objective and laser excitation wavelengths of 405, 488, 561 and 633 nm. Some samples were imaged using a structured illumination microscopy (N-SIM, Nikon) using an iXon3 EM-CCD camera (Andor), respectively. Images within the same experiments were all acquired at the same laser settings using Nikon NIS Elements software.

### G-LISA analysis

Cdc42 activation analysis from cell lysates was carried out using G-LISA assays (cytoskeleton) according to manufacturers’ instructions. Each experiment was performed in triplicate.

### FRET biosensor imaging and analysis

Cells were transiently transfected with Cdc42 or RhoGDI biosensors. FRET imaging was carried out at 37°C using a TiE 2 wide-field fluorescence microscope (Nikon). Images were acquired using ECFP/EYFP FRET filter set (Chroma 89002) and 40× oil objective. Three images were captured simultaneously using an EMCCD camera: CFP channel image (CFPex-CFPem), YFP channel image (YFPex-YFPem) and FRET channel image (CFPex-YFPem). Bleed-through was corrected for using CFP-only and YFP-only expressing lines. Ratiometric analysis was carried out between the YFP and FRET channel images using the ImageJ plugin RatioPLUS. 25-pixel rolling background subtraction was applied to both images. A background region of interest (ROI) was selected and measured in both channels. A cell with a signal intensity that represents the majority of the population was selected, and its minimum intensity was measured. The clipping value was calculated for both channels, which along with the background signal, was entered into the RatioPLUS plugin. The same clipping value was used for all the fields of view in the same experiment to make ratiometric images comparable. Following the application of the RatioPLUS plugin a 16-colour look up table (LUT) was applied. Outlier pixel values were removed from the ratio image using the built in ImageJ plugin Remove Outliers (radius=2, threshold=50), and a 3D median blur (x=1, y=1, z=1) was applied to smooth the image.

### Wound healing assays

Control or α2-integrin knockdown/rescue keratinocytes were grown to confluency in 12-well culture plates in the presence of 2mM calcium. A wound was created using a 10-μm pipette tip, and cells were imaged using an EVOS2 microscope platform (Thermofisher). Wound healing assays were analysed using ICY. The wound area was manually measured for each time point using the polygon tool. The closure was measured compared to time point 0.

### Junction intensity analysis

Control or α2-integrin knockdown cells transiently expressing LifeAct-GFP were plated in 12-well tissue culture plates and grown to confluency. The media was then exchanged for media containing with or without 2mM CaCl_2_ or or MgCl_2_ and either DMSO, BTT3033 or ML141 for the duration of the experiment. For junctional intensity quantification from fixed images, junctions were identified through markers (E-cadherin, F-actin as specified). In ICY, one ROI was drawn of constant width along the junctions and a second in the cell body. The intensity/μm^2^ was measured (background subtracted using the cell body ROI) and compared for the different conditions. For line scan analysis, in FIJI 10×1 μm lines were drawn perpendicular to the junctions. The 5-μm point marked the centre of junctions and intensity was exported and the individual line scans were normalised to control.

### Western blotting, immunoprecipiation and GFP trap

Cells were lysed in sample buffer containing β-mercaptoethanol at room temperature. Lysates were subjected to SDS–polyacrylamide gel electrophoresis (PAGE) and blotted using PVDF membrane. Blots were blocked and probed using 5% bovine serum albumin (BSA)/PBS–0.1% Tween 20 and quantified using ECL Plus Western blot detection system (GE Healthcare). For GFP trap experiments, cells expressing either GFP or α2-integrin-GFP were lysed in lysis buffer (50 mM tris (pH 7.4), 200 mM NaCl, 2 mM MgCl_2_, 1% NP-40, 10% glycerol, and protease inhibitor cocktail). Lysates were incubated with 5 μg of GFP-antibody pre-bound to A/G agarose beads overnight before washing the beads with 1 ml of IP lysis buffer three times. For IP experiments, cells were lysed in lysis buffer (50 mM tris (pH 7.4), 150 mM NaCl, 1 mM EDTA, 1% Triton, and protease inhibitor cocktail) and incubated with 3 μg of either primary antibody or an IgG control. Immuno-complexes were separated using SDS-PAGE and immuno-blotted for specified proteins.

### Proliferation analysis

1×10^4^ control or α2-integrin knockdown cells were plated into 24-well plates and incubated for 24, 48 or 72 h. Hoechst was added to the medium of the cells for 30 min to stain DNA and then cells were fixed for 15 min in 4% PFA/PBS in the dark. Analysis was performed on an EVOS FL Auto 2 (Invitrogen) after staining. Cells were imaged using the same exposure and images of the total well were saved as TIFF files. The images were analysed by automatically counting the number of nuclei in each condition using a custom-made FIJI macro. Filopodia analysis was conducted using CellGeo, a MATLAB application [62].

### Ligand adhesion assays

Coverslips were coated with either 200μg/ml Fc-E-cadherin ectodomain, 10μg/ml laminin5 (Abcam ab42326) or 50μg/ml collagen (C4243; Merck). WT, control or α2-integrin knockdown NHKs were plated, either untreated or in the presence of DMSO, BTT3033 or ML141, with or without 2mM CaCl_2_. Cells were left to adhere for 30 min and then fixed, stained and imaged as previously described.

### Statistical analysis

All statistical tests were performed using Students T tests or ANOVA (GraphPad, Prism). Significance values are assigned in specific experiments.

## Supplementary Information

**Additional file 1: Figure S1.** (a) Images of WT monolayers with 2mM Ca^2+^ fixed and stained for F-actin and α3, α3 or α5 integrin subunits and β1 integrin. Scale bars, 10μm. (b) Images of WT monolayers +/- 2mM Ca^2+^ fixed and stained for F-actin and active β1 integrin. Quantification of relative junctional intensities of total and active β1 integrin without and with 2mM Ca^2+^ from 35 cells per condition; representative of 3 independent experiments. Scale bars, 10μm. (c) Images of WT monolayers with 2mM Mg^2+^ +/- Ca^2+^ fixed and stained for F-actin and active β1 integrin. Quantification of relative junctional intensities of active β1 integrin without and with 2mM Mg^2+^ or Ca^2+^ from 30 cells per condition; representative of 3 independent experiments. Scale bars, 10μm. (d) Western blot analysis of cells for integrin subunits α2, α3, α5 and β1 or actin, E-cadherin and β-catenin with GAPDH as a loading control. (e) Representative confocal images of Control and α2KD cells or WT cells treated with DMSO or BT fixed and stained for F-actin and active β1 integrin. Scale bars, 10μm. (f) Representative confocal images of WT cells treated with DMSO or BTT (20 μm, 1hr) fixed and stained for F-actin and E-cadherin and quantification of E-Cadherin and F-actin intensity at junctions from 30 cells per condition from 3 independent experiments. Scale bars 10μm. (g) Confocal slices from junctional and basal planes of Control and α2KD monolayers in 2mM Ca^2+^ fixed and stained for F-actin and vinculin and quantification of vinculin positive focal adhesion at basal planes from 35 cells per condition; representative of 3 independent experiments. Scale bars, 10μm. (h) Analysis of cell monolayer permeability in WT, Control, α2 knockdown (KD1 and KD2) and α2KD1 cells re-expressing α2-GFP following 2 hours of fluorescent dextran incubation. 1mM EDTA was used a positive control. Data is from *n*=4 wells per condition, and representative of 3 independent experiments. (i) Analysis of proliferation of WT, Control and α2 knockdown (KD1 and KD2) and KD1 cells stably rescued with α2-GFP over 72h under normal growth conditions. *n*=4 wells per condition; representative of 3 independent experiments. (j) Quantification of % wound closure from 24h movies of WT, Control, α2 knockdown (KD1 and KD2) and α2KD1 cells re-expressing α2-GFP. *n*=3 wells per condition; representative of 3 independent experiments. *** *p*<0.001, **p<0.01, *p<0.05.

**Additional file 2: Figure S2.** a) Quantification of the percentage of cells adhered onto collagen, LN or Fc-E-cadherin following 60 minutes incubation, representative of 3 independent experiments. (b) Representative image of control cells plated onto Fc-E-cadherin coated coverslip for 30 minutes and fixed and stained for α2 integrin and E-cadherin. Scale bar 10μm. (c) Confocal images of basal plane of WT monolayers in 2mM Ca^2+^, fixed and stained for DAPI, laminin α3 and F-actin. Scale bars 10μm. *** *p*<0.001, *p<0.05. (d) Representative confocal images of human skin sections stained for α2 integrin, laminin α3, Laminin β1 or Collagen IV. Bottom panel shows zoomed images of example regions where Laminin interdigitates between keratinocytes. Scale bars 10μm.

**Additional file 3: Figure S3. (**a) Images of control and α2 knockdown (KD) cells treated with either DMSO or ML141 (10 μm, 4h) and fixed and stained for DAPI and E-cadherin. Scale bars, 10μm. (b) Quantification of E-cadherin intensity at junctions and junction width from images as in (a). (c) Representative blots of lysates from α2KD cells expressing GFP or α2-GFP with or without 2mM Ca^2+^ (- and + respectively), immunoprecipitated with GFP antibodies and complexes probed for α2, Cdc42 or GFP. Input levels are shown on the left. (d) Representative blots of lysates from α2KD cells expressing GFP or α2-GFP with or without 2mM Ca^2+^ (- and + respectively), immunoprecipitated with GFP antibodies and complexes probed for α2, IQGAP1, RhoGDI, RacGAP1 or Tuba. Input levels are shown on the left. (e) Images of DMSO and BTT treated cells fixed and stained for pY156 RhoGDI and E-Cadherin; quantification of images from at least 30 images per condition over 3 independent experiments. Scale bars, 10μm. (f) GFP trap of lysates from WT cells expressing either GFP or RhoGDIα-GFP treated with DMSO or PP2 (10 μm, 1hr). Complexes from GFP traps were probed for phosphotyrosine (PY) and GFP. (g) GFP trap of lysates from WT cells expressing either GFP or RhoGDIα-GFP treated with Ca^2+^ (2mM) for 5 mins. Complexes from GFP traps were probed for phosphotyrosine (PY) and GFP. ***= *p*<0.001, **= *p*<0.01, *= *p*<0.05.

**Additional file 4: Figure S4.** (a) Images of Control and α2 knockdown (KD) monolayers in Ca^2+^, fixed and stained for p-Src and E-cadherin. Scale bars 10μm. (b) Images of Control and α2 knockdown (KD) monolayers in Ca^2+^, treated with either DMSO or PP2 (10 μm, 1hr), fixed and stained for E-cadherin. Scale bars 10μm.

**Additional file 5.** Full blots for all data shown in Figs. 1, 2, 3, 4 and Additional Files 1, 2, 3, 4.

## Data Availability

All data generated or analysed during this study are included in this article and its supplementary information files. Plasmids are available from the corresponding author upon request.
