# Supplementary figures and images for: α2β1 integrins spatially restrict Cdc42 activity to stabilise adherens junctions
Source: BMC Biol. 2021 Jun 23;19:130. doi: 10.1186/s12915-021-01054-9 (PMC8220754; doi:10.1186/s12915-021-01054-9)

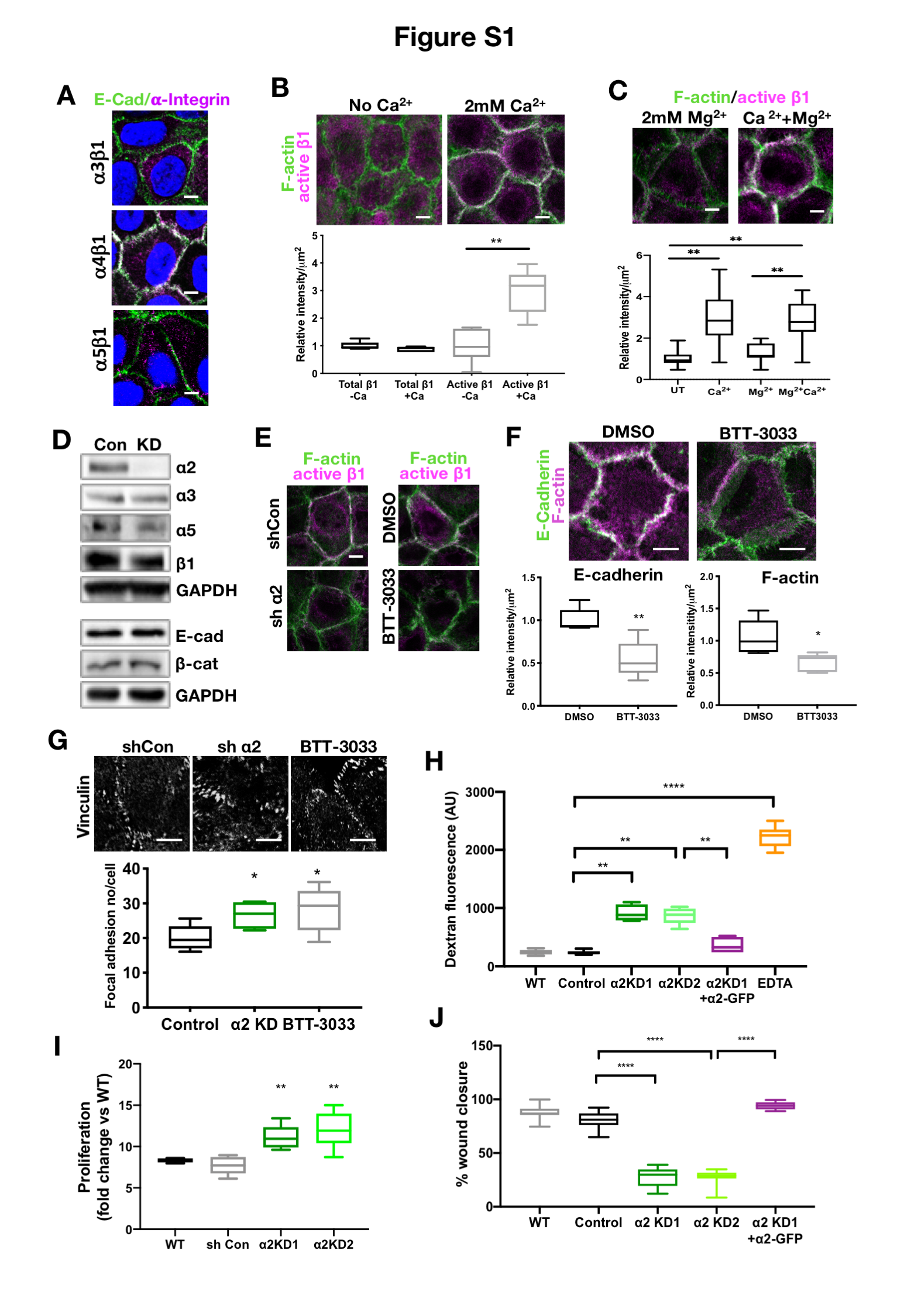

Supplement: Supplementary file 1 — Additional file 1: Figure S1. (a) Images of WT monolayers with 2mM Ca2+ fixed and stained for F-actin and α3, α3 or α5 integrin subunits and β1 integrin. Scale bars, 10μm. (b) Images of WT monolayers +/- 2mM Ca2+ fixed and stained for F-actin and active β1 integrin. Quantification of relative junctional intensities of total and active β1 integrin without and with 2mM Ca2+ from 35 cells per condition; representative of 3 independent experiments. Scale bars, 10μm. (c) Images of WT monolayers with 2mM Mg2+ +/- Ca2+ fixed and stained for F-actin and active β1 integrin. Quantification of relative junctional intensities of active β1 integrin without and with 2mM Mg2+ or Ca2+ from 30 cells per condition; representative of 3 independent experiments. Scale bars, 10μm. (d) Western blot analysis of cells for integrin subunits α2, α3, α5 and β1 or actin, E-cadherin and β-catenin with GAPDH as a loading control. (e) Representative confocal images of Control and α2KD cells or WT cells treated with DMSO or BT fixed and stained for F-actin and active β1 integrin. Scale bars, 10μm. (f) Representative confocal images of WT cells treated with DMSO or BTT (20 μm, 1hr) fixed and stained for F-actin and E-cadherin and quantification of E-Cadherin and F-actin intensity at junctions from 30 cells per condition from 3 independent experiments. Scale bars 10μm. (g) Confocal slices from junctional and basal planes of Control and α2KD monolayers in 2mM Ca2+ fixed and stained for F-actin and vinculin and quantification of vinculin positive focal adhesion at basal planes from 35 cells per condition; representative of 3 independent experiments. Scale bars, 10μm. (h) Analysis of cell monolayer permeability in WT, Control, α2 knockdown (KD1 and KD2) and α2KD1 cells re-expressing α2-GFP following 2 hours of fluorescent dextran incubation. 1mM EDTA was used a positive control. Data is from n=4 wells per condition, and representative of 3 independent experiments. (i) Analysis of proliferation of WT, Co [file 12915_2021_1054_MOESM1_ESM.png]

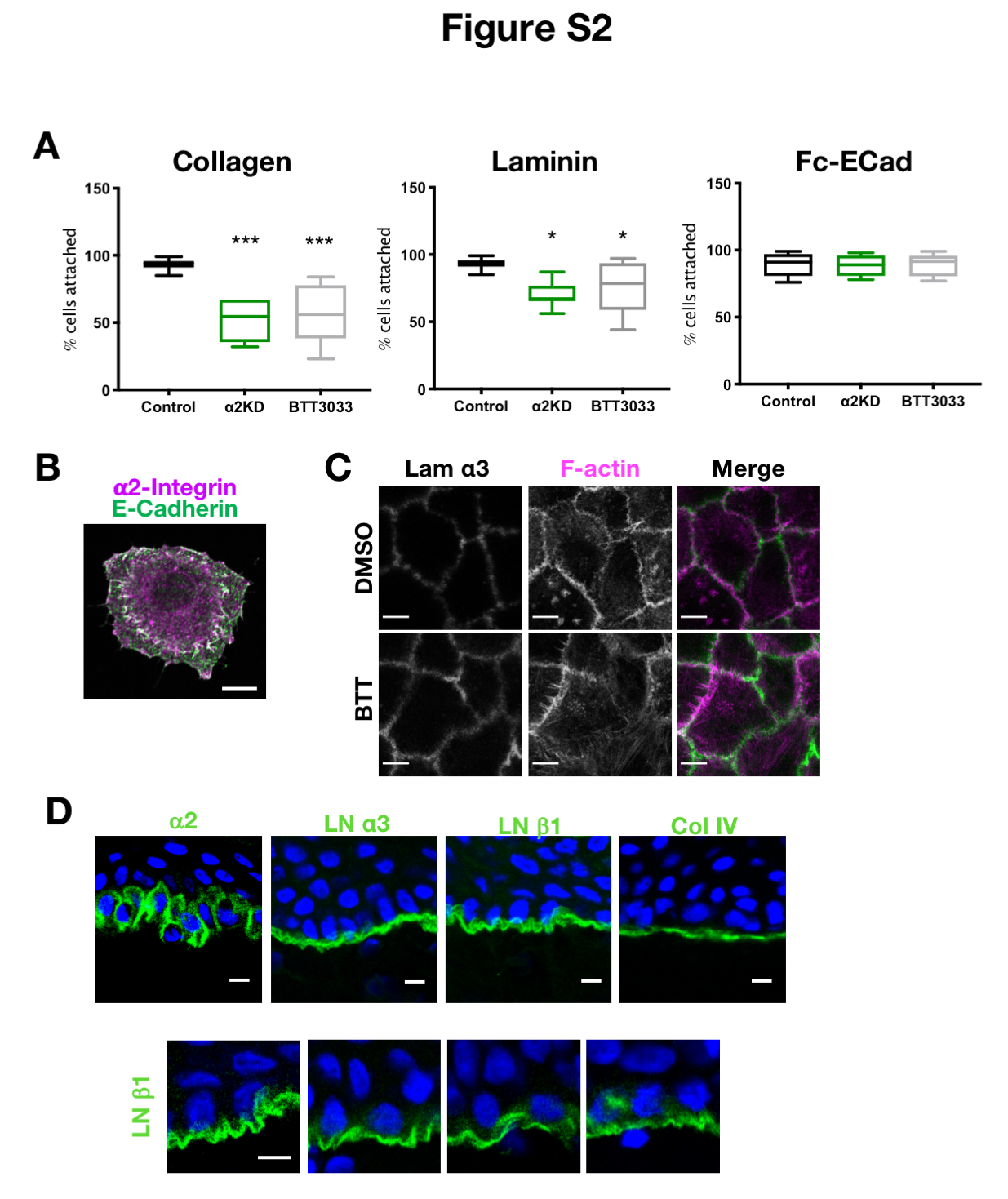

Supplement: Supplementary file 2 — Additional file 2: Figure S2. a) Quantification of the percentage of cells adhered onto collagen, LN or Fc-E-cadherin following 60 minutes incubation, representative of 3 independent experiments. (b) Representative image of control cells plated onto Fc-E-cadherin coated coverslip for 30 minutes and fixed and stained for α2 integrin and E-cadherin. Scale bar 10μm. (c) Confocal images of basal plane of WT monolayers in 2mM Ca2+, fixed and stained for DAPI, laminin α3 and F-actin. Scale bars 10μm. *** p<0.001, *p<0.05. (d) Representative confocal images of human skin sections stained for α2 integrin, laminin α3, Laminin β1 or Collagen IV. Bottom panel shows zoomed images of example regions where Laminin interdigitates between keratinocytes. Scale bars 10μm. [file 12915_2021_1054_MOESM2_ESM.tiff]

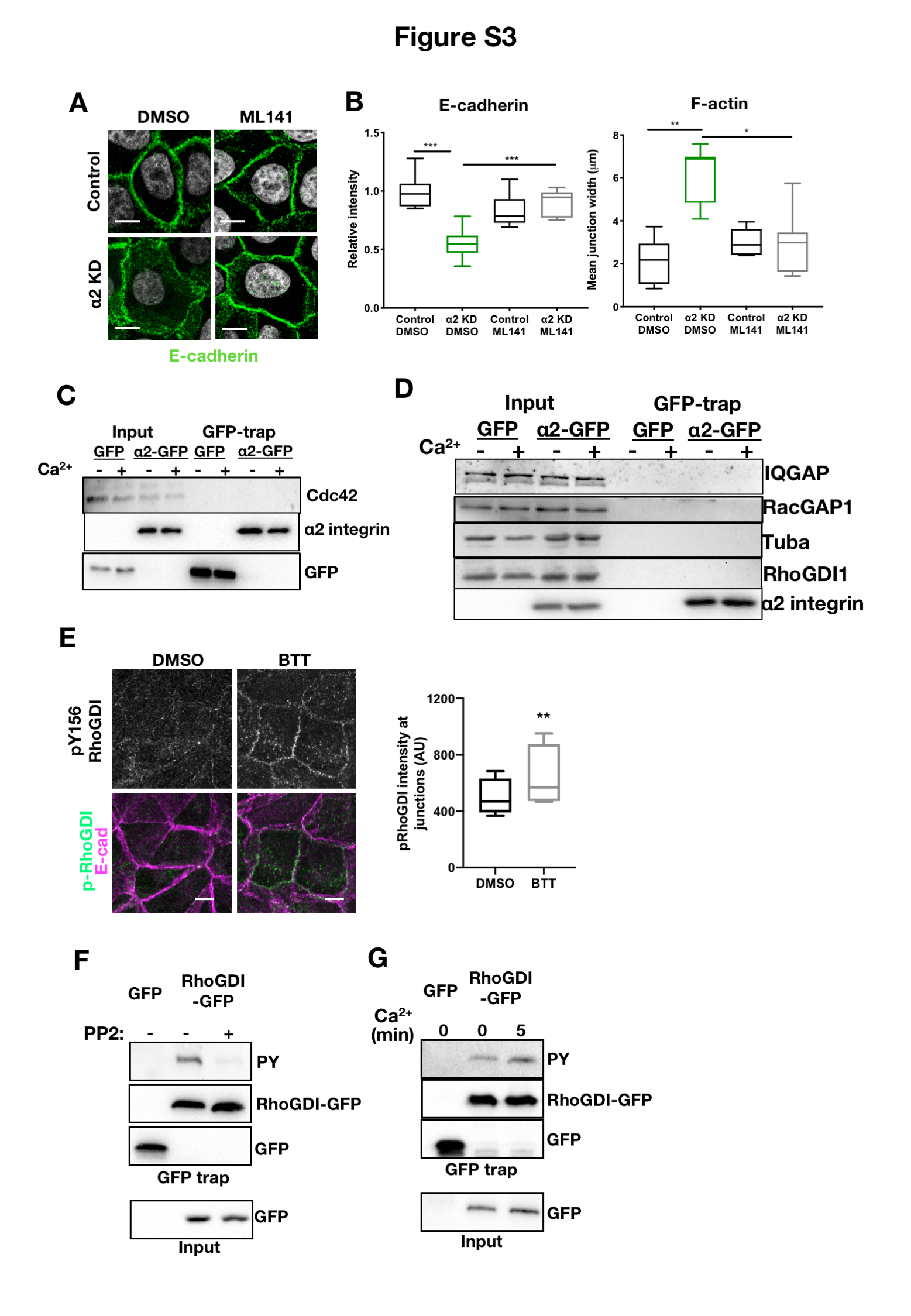

Supplement: Supplementary file 3 — Additional file 3: Figure S3. (a) Images of control and α2 knockdown (KD) cells treated with either DMSO or ML141 (10 μm, 4h) and fixed and stained for DAPI and E-cadherin. Scale bars, 10μm. (b) Quantification of E-cadherin intensity at junctions and junction width from images as in (a). (c) Representative blots of lysates from α2KD cells expressing GFP or α2-GFP with or without 2mM Ca2+ (- and + respectively), immunoprecipitated with GFP antibodies and complexes probed for α2, Cdc42 or GFP. Input levels are shown on the left. (d) Representative blots of lysates from α2KD cells expressing GFP or α2-GFP with or without 2mM Ca2+ (- and + respectively), immunoprecipitated with GFP antibodies and complexes probed for α2, IQGAP1, RhoGDI, RacGAP1 or Tuba. Input levels are shown on the left. (e) Images of DMSO and BTT treated cells fixed and stained for pY156 RhoGDI and E-Cadherin; quantification of images from at least 30 images per condition over 3 independent experiments. Scale bars, 10μm. (f) GFP trap of lysates from WT cells expressing either GFP or RhoGDIα-GFP treated with DMSO or PP2 (10 μm, 1hr). Complexes from GFP traps were probed for phosphotyrosine (PY) and GFP. (g) GFP trap of lysates from WT cells expressing either GFP or RhoGDIα-GFP treated with Ca2+ (2mM) for 5 mins. Complexes from GFP traps were probed for phosphotyrosine (PY) and GFP. ***= p<0.001, **= p<0.01, *= p<0.05. [file 12915_2021_1054_MOESM3_ESM.png]

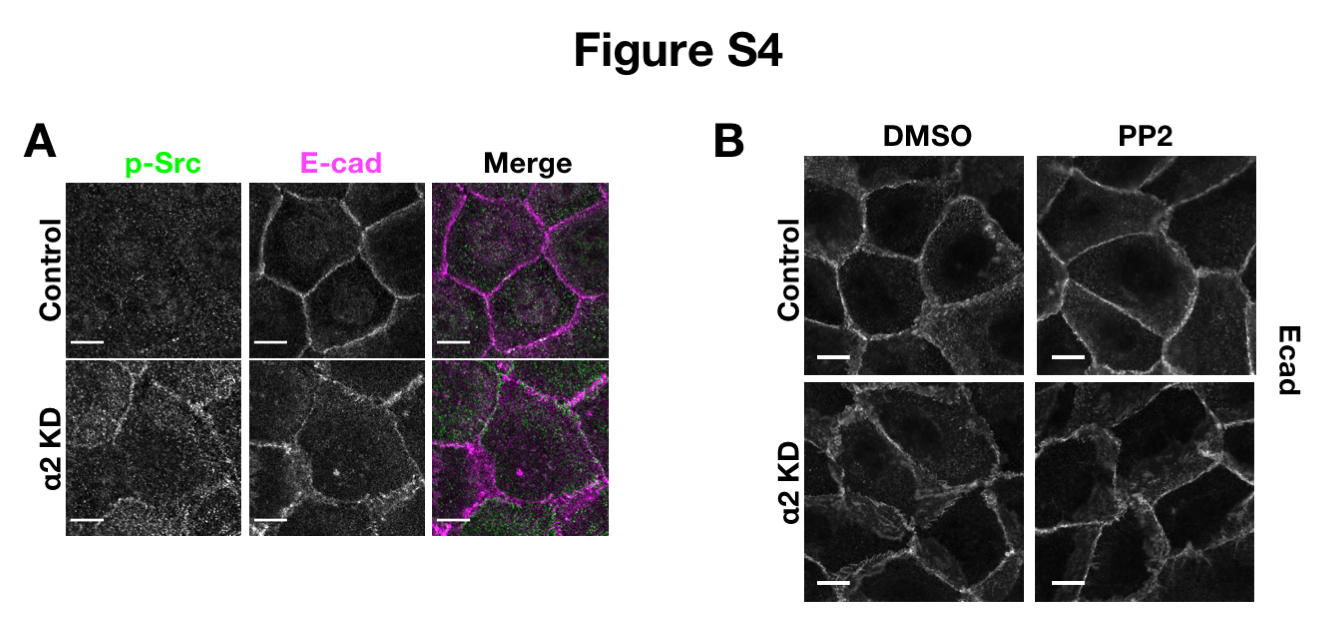

Supplement: Supplementary file 4 — Additional file 4: Figure S4. (a) Images of Control and α2 knockdown (KD) monolayers in Ca2+, fixed and stained for p-Src and E-cadherin. Scale bars 10μm. (b) Images of Control and α2 knockdown (KD) monolayers in Ca2+, treated with either DMSO or PP2 (10 μm, 1hr), fixed and stained for E-cadherin. Scale bars 10μm. [file 12915_2021_1054_MOESM4_ESM.tiff]

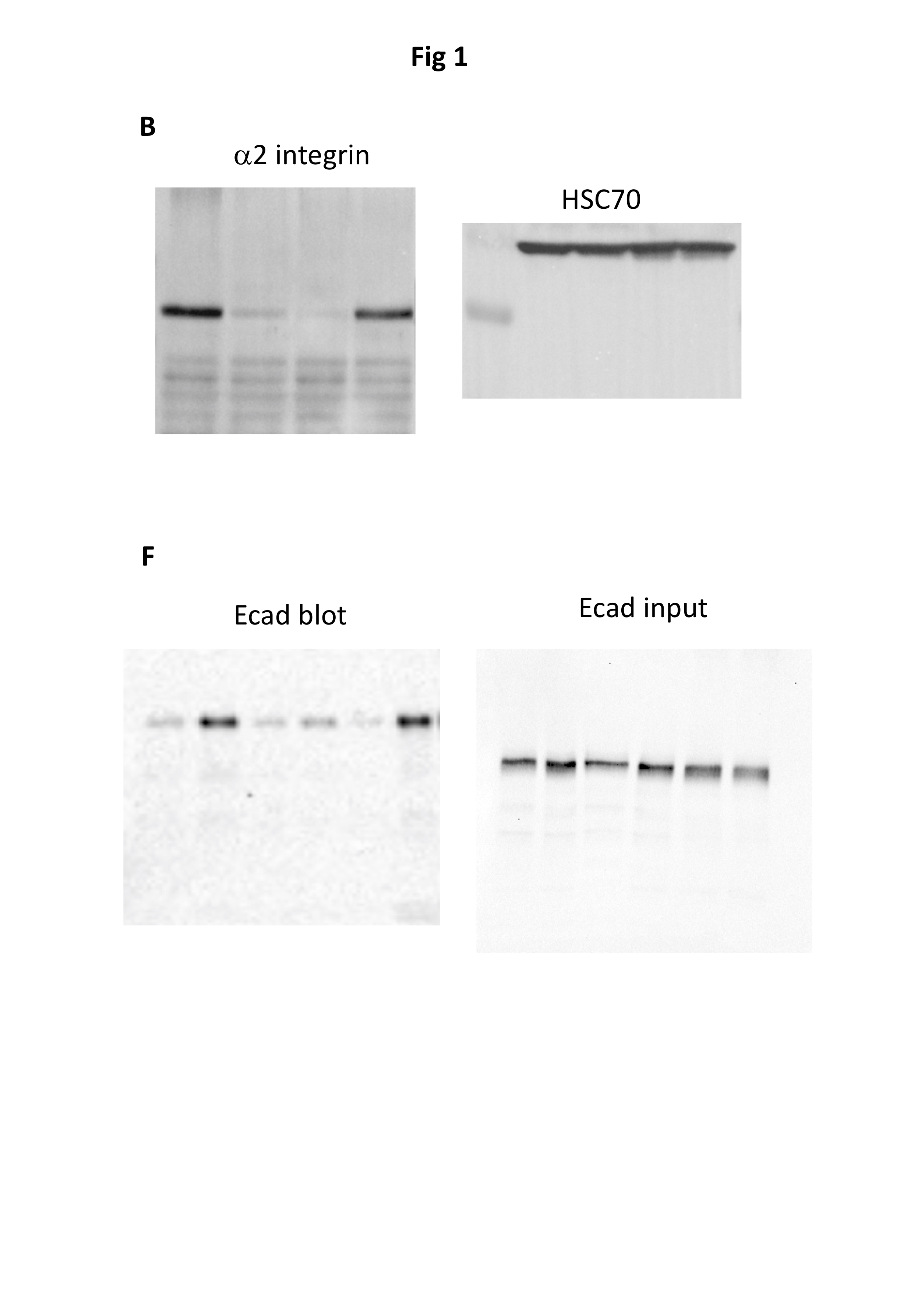

Supplement: Supplementary file 5 — Additional file 5. Full blots for all data shown in Figs. 1, 2, 3, 4 and Additional Files 1, 2, 3, 4. [file 12915_2021_1054_MOESM5_ESM.jpg]
